# AI-Powered Innovations in Food Safety from Farm to Fork

**DOI:** 10.3390/foods14111973

**Published:** 2025-06-02

**Authors:** Binfeng Yin, Gang Tan, Rashid Muhammad, Jun Liu, Junjie Bi

**Affiliations:** 1 School of Mechanical Engineering, Yangzhou University, Yangzhou 225127, China; creatgang@163.com (G.T.); chemistwindow@gmail.com (R.M.); 18205073638@163.com (J.B.); 2 Suqian Product Quality Supervision and Inspection Institute, Suqian 223800, China; ljljljlj1982@163.com

**Keywords:** AI, food safety, from farm to fork, full chain, rapid detection

## Abstract

Artificial intelligence is comprehensively transforming the food safety governance system by integrating modern technologies and building intelligent control systems that provide rapid solutions for the entire food supply chain from farm to fork. This article systematically reviews the core applications of AI in the orbit of food safety. First, in the production and quality control of primary food sources, the integration of spectral data with AI efficiently identifies pest and disease, food spoilage, and pesticide and veterinary drug residues. Secondly, during food processing, sensors combined with machine learning algorithms are utilized to ensure regulatory compliance and monitor production parameters. AI also works together with blockchain to build an immutable and end-point traceability system. Furthermore, multi-source data fusion can provide personalized nutrition and dietary recommendations. The integration of AI technologies with traditional food detection methods has significantly improved the accuracy and sensitivity of food analytical methods. Finally, in the future, to address the increasing food safety issues, Food Industry 4.0 will expand the application of AI with lightweight edge computing, multi-modal large models, and global data sharing to create a more intelligent, adaptive and flexible food safety system.

## 1. Introduction

Food safety is a core issue worldwide and directly related to human health [1,2], social stability [3,4], and economic development. According to the World Health Organization, approximately 600 million people are affected every year by eating contaminated food, of which 420,000 die [5,6,7,8]. Food safety is an important public health issue that has affected all stages of the food industrial chain [9], such as the repurposing of spoiled food [10,11], the fraudulent use of additives in processing [12,13], the excessive use of preservatives [14,15], the return of cooking oil to the table [16], the illegal processing of lymph meat [17], highly toxic packaging [18,19], and low-quality meat production [20,21]. Long-term intake of such food can damage the liver, kidneys, and other organs; similarly, these contaminated foods can also cause different cardiovascular diseases, metabolic diseases, and even cancer [22,23,24]. Food safety issues arise because some industries try to make extra profit, engage in malicious practices, and exploit regulatory loopholes. Thus, to maintain standards, there is a need to improve regulations and standards, strictly control the source of production [25,26], upgrade the testing system, strengthen the traceability mechanism, and popularize nutrition labeling, among other actions, thereby building a whole chain of security defense from farm to fork [27,28,29,30].

With the complexity of the global food supply chain and consumer demand for transparency, food safety management has shifted from the detection of contamination at the end to whole-chain risk prevention and control [31,32,33]. After Norman Borlaug’s “Green Revolution”, mechanization and large-scale food production and processing improved the quality and quantity of food production, but the risks such as the abuse of chemical fertilizers [34,35] and pesticides [36,37,38], excessive veterinary drug residues [39,40,41], and microbial pollution [42] have increased exponentially [43]. To detect these poisons and contamination, different analytical methods have been developed. These detection methods rely on manual sampling [44,45] and laboratory analysis [46,47,48] and have limitations like poor timeliness, low coverage rates, and tedious analytical procedures. Secondly, it is difficult to achieve on-site detection using existing detection technology [49] and real-time monitoring [50,51,52], and there are still limitations in the integration, intelligence, and convenience of detection technology [53,54,55]. These obstacles greatly reduce the efficiency of food detection technology. The gap between food production development and testing technology makes the prevention and control of food safety accidents more challenging [56,57,58,59].

**Figure 1:**
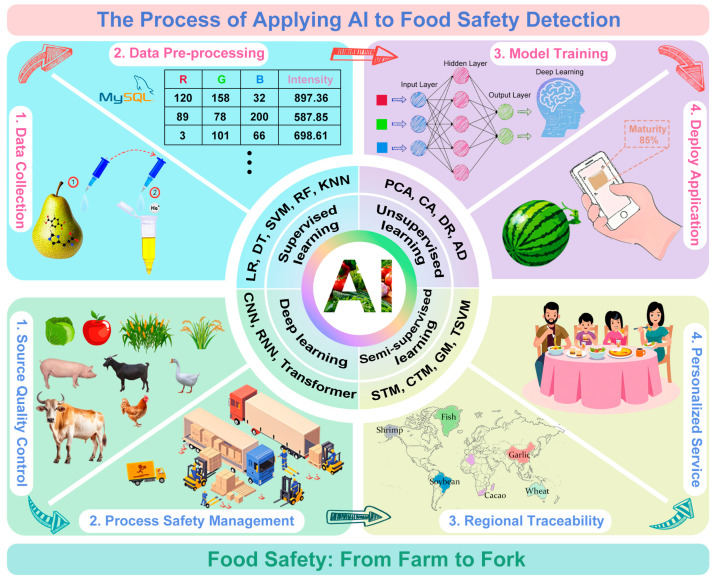
General process of Al applied to food safety detection. Specific applications of AI in 1. source quality control, 2. process safety management, 3. regional traceability, and 4. personalized service.

The development of AI technology for food safety provides a revolutionary tool for restructuring the food safety governance system [60,61,62]. By integrating cutting-edge technologies such as spectral analysis [63,64], machine vision [32,65], sensor networks [66,67,68], and blockchain [69,70], AI can be used to build a full-chain intelligent management and control system covering the whole process from farm to fork. As shown in Figure 1, the application of AI in food source quality control, process safety management, regional traceability, and personalized service is very diverse and demonstrates great potential. In agricultural production, the pest and disease identification system based on deep learning (DL) and big data analysis has realized the real-time monitoring of more than 200 diseases. In the processing system, the fusion technology of hyperspectral imaging and machine learning (ML) can accurately detect any illegal food additives and adulteration. At the consumer end, smartphone-based colorimetric sensors combined with edge computing models enable consumers to identify meat freshness. These technological innovations not only overcome the limitations of traditional detection methods for food contaminants but also change the data- and experience-dependent decision-making system to enable intelligent forecasting for food safety [71,72,73,74].

This article systematically reviews the core applications of AI in the orbit of food safety. First, in the production and quality control of primary food sources, the integration of spectral data with AI efficiently identifies pests and diseases, food spoilage, and pesticide and veterinary drug residues. Secondly, during food processing, sensors combined with machine learning algorithms are utilized to ensure regulatory compliance and monitor production parameters. AI also works together with blockchain to build an immutable and end-point traceability system. Furthermore, multi-source data fusion can provide personalized nutrition and dietary recommendations. This study highlights how AI-driven algorithm models can be combined with various detection technologies, such as spectroscopy, machine vision, big data, and sensors, to address the technical barriers encountered by traditional detection technologies in food safety. Additionally, it systematically summarizes the technological breakthroughs of AI in the field of food safety and constructs an interdisciplinary research framework. In multidisciplinary fields such as food science, computational chemistry, and operations research, AI technology can create a new governance system through integration. AI-driven food safety governance is improving from technical exploration to institutional innovation worldwide, which marks a new era of intelligent governance in the effort to ensure food safety.

## 2. AI-Based Food Detection Technology

### 2.1. Literature Search and Screening Methods

Based on a search of the Web of Science, X-MOL, and IEEE Xplore core databases, this study identified 1528 relevant papers published up until April 2025, focusing on the keywords “artificial intelligence”, “machine learning”, “neural network”, and “food safety”. Following the order from farm to fork, this review systematically analyzed innovative development brought by the combination of AI technologies and traditional detection methods in the field of food safety. The search strategy was determined mainly through the following seven aspects, with the application scenario and detection purposes used as the inclusion and exclusion criteria. For food source management in farming, we mainly focused on the application of AI technology in crop pest management, pesticide and fertilizer residues, veterinary drug residues, heavy metal pollution, and crop planting structure. In food quality screening, spectral data and machine learning algorithms are used to screen foreign substances and defects and assess meat freshness. In food storage monitoring, the integration of the internet of things and AI technologies is used to monitor the storage environment in real time, analyzing the risk of food spoilage and the quality of perishable and difficult-to-store foods. In quality control in food processing, deep learning and machine vision are used to monitor production parameters in real time and strictly supervise the quality and safety of food packaging. In the detection of contamination in food products, the integration of AI technologies and the colorimetric method is used for the rapid detection of food quality. In food traceability, a trusted traceability architecture of blockchain +AI builds tamper-resistant food information. Personalized meal services for consumers provide information on the influence of healthy food on human diseases and special proteins and balanced meals for customized nutrition. The first six aspects ensure the absolute safety of food placed on the table and the seventh aspect aims to solve the contradiction between healthy eating and personalized nutrition needs.

**Figure 2:**
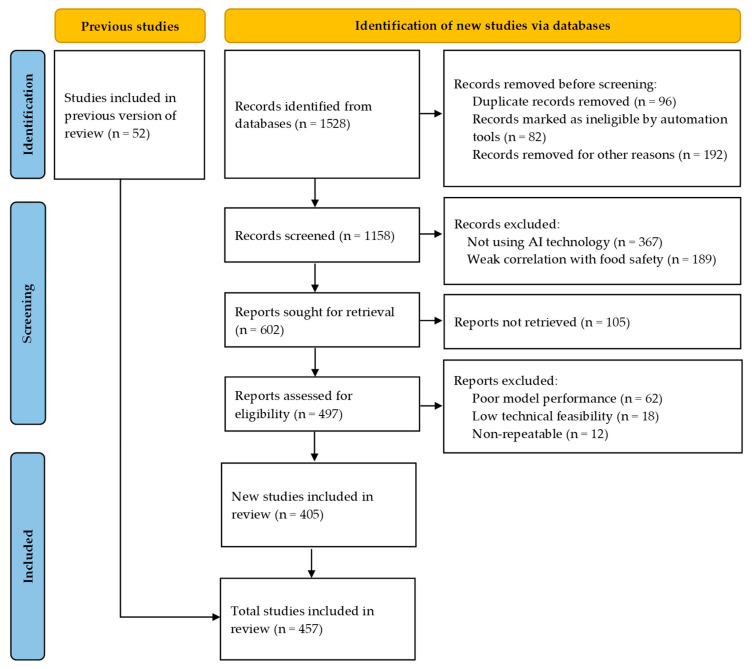
The flow chart of PRISMA.

For the 1528 retrieved studies, we used seven application scenarios from farm to fork, including factors such as detection purpose, technical feasibility, model performance, innovation, and repeatability, in the quality assessment framework. Based on this quality assessment, we selected 276 high-quality studies, 129 medium-quality studies, and 1123 low-quality studies. The low-quality literature was not included in the scope of this study. The specific literature search following the PRISMA process is shown in Figure 2.

### 2.2. Background of AI Applications in Food Safety

As an emerging technology in the new era of the industrial revolution, AI is changing the orientation of traditional industries. One important factor is AI’s role in food safety [75,76]. The rise of AI in the field of food safety is the result of technological innovation, industrial upgrading, market demand, the search for healthy food, timely and cost-effective analytical methods, and the demand for green and high-quality products [77,78]. In the whole chain management system of food safety, for the accurate control of food sources through intelligent sorting before pre-processing, food storage safety specifications, the strict supervision of food processing, food safety testing, blockchain and AI trusted traceability architecture, and personalized intelligent services, the traditional supervision model has obvious defects in detection methods, efficiency, cycle and accuracy. In terms of efficiency, manual sampling is time-consuming and laborious, resulting in a prolonged detection cycle and response to food safety risks. In terms of traceability, due to limited detection samples and a lack of systematic records, when defective food is found, it is extremely difficult to trace its source. In terms of coverage, most manual sampling is not truly representative, and in the processing and circulation stages, there are many regulatory blind spots [49,79,80]. More importantly, in the whole industry chain of food from farm to fork, traditional detection methods are unable to achieve real-time and continuous monitoring or detect any delicate changes that may occur in the production, processing, storage, and transportation of food [81,82,83].

Nowadays, food safety detection remains a thorny issue; the adulteration of food is increasing and it is difficult to detect adulteration at every stage. This is due to the high analytical cost, long analysis time, and lack of resources required for these sophisticated techniques [84]. To resolve this complex issue in food safety and improve detection efficiency and accuracy, AI has brought new possibilities with its powerful data processing capabilities, efficient automation characteristics, and accurate analysis and judgment [85]. AI technology, including computer vision, the internet of things, natural language processing, and DL, can achieve real-time data acquisition, automated defect detection, component analysis, and risk prediction. By combining blockchain with AI, the whole process can be monitored to achieve traceability. AI can also improve the coordinated governance of governments, enterprises, consumers, and farmers by building an intelligent prevention and control system. This will build a full chain security system from farm to fork, covering production, circulation, and consumption. In the future, with the integration of high-speed communication and digital twins, AI will shift from a single point of application to a systematic restructuring of food safety governance models [27,86,87].

### 2.3. Classification of AI Algorithms in Food Monitoring

AI technology builds intelligent systems by simulating human cognitive mechanisms and completes some complex tasks, even surpassing human ability. Its core technologies include computing power architecture, high-quality labeled data, and ML algorithms. AI technology has revolutionized food safety, quality control, and identification systems with automated analysis. This enables analysts to accurately identify contaminants and make real-time decisions [88]. AI provides intelligent solutions for food detection with minimal errors. According to different learning modes, ML can be divided into three core branches: supervised learning, unsupervised learning, and semi-supervised learning. DL, as an important branch of ML, belongs to the categories of supervised learning and unsupervised learning, also known as deep neural networks (DNNs) [89,90,91].

#### 2.3.1. Supervised Learning

Supervised learning generally includes linear regression (LR), logistic regression, decision tree (DTs) [92,93], support vector machines (SVMs) [94,95], random forest (RF) [96,97], and K-nearest neighbor (KNN). Its core feature is to use labeled data to train classification or regression models and predict or classify unknown data by learning input and output mapping relationships [98]. In food safety, supervised learning can be used for food quality classification and defect detection, microbial contamination prediction [99], food adulteration and composition identification, and toxic substance detection. Through automated and high-precision data analysis, food detection efficiency, risk prevention, and control ability are significantly improved. Rong et al. [100] proposed a two-stage convolutional neural network (CNN) solution based on DL to solve the problems of the low efficiency of traditional walnut impurity detection methods. Image segmentation and impurity identification were realized by a multiscale, residual, fully convolutional network and a four-class CNN. The detection problems in complex scenarios, such as adhesion between walnut and impurities and surface wear interference of conveyor belts, were successfully resolved. This method achieved 99.4% section segmentation accuracy and 96.1% impurity detection accuracy for the test image, and the processing time of a single image was less than 60 ms, which showed significant improvement in the sorting of impurities in walnuts.

#### 2.3.2. Unsupervised Learning

Unsupervised learning generally includes principal component analysis (PCA) [101,102,103], K-means clustering (K-means) [104,105], cluster analysis (CA) [106], dimension reduction (DR) technology, and anomaly detection (AD). Unsupervised learning can independently discover hidden patterns or anomalies in unlabeled data, which is suitable for exploratory data analysis and complex scene modeling. Unsupervised learning can be applied to anomaly detection, defect detection, food adulteration, and ingredient abnormalities in food production and the supply chain. The value of unsupervised learning in the field of food safety lies in proactively discovering unknown risks and ensuring timely prevention and supervision. Chen et al. [107] designed a real-time detection device and method based on machine vision for the quality of metal cans in the manufacturing process, which was used to determine the integrity and safety of food packaging. The device used a multi-stage algorithm and a specific imaging scheme to solve the problem of complex surface illumination and could efficiently and accurately identify a variety of defects to ensure the safety of food packaging. The experimental results showed that the system could detect the round bottom of different sizes with an accuracy of 99.48%, and the processing time of a single bottom of the tank was 0.7 s. The system could effectively identify eight types of typical defects and provide a reliable solution for food packaging safety.

#### 2.3.3. Semi-Supervised Learning

Semi-supervised learning generally includes the self-training model (STM), co-training model (CTM), generative model (GM), and transduction SVM (TSVM) learning model. Semi-supervised learning combines supervised learning and unsupervised learning, using a small amount of labeled data to train a model and mining the distribution characteristics of a large amount of unlabeled data to enhance the generalization ability of the model and reduce the cost of data labeling. In practical applications, semi-supervised learning can reduce the need for data labeling while maintaining detection accuracy, which is suitable for identifying food appearance defects, component analysis, foreign body detection, and supply chain risk prediction. Looverbosch et al. [108] used X-ray computed tomography combined with ML technology for the non-destructive testing of pear fruit internal quality, testing for browning, cavities, and other defects during long-term storage. This resolved the limitations of low sensitivity of traditional spectral detection methods. In pear fruit quality detection, SVM was used as a classifier to construct an automatic detection system. A few 3D mask fruit samples of labeled fruit were combined with unlabeled fruit data to determine the quality of fruit. The overall accuracy of the method was 92.2% in the five-fold cross-validation. This ML-based, 3D, nondestructive testing scheme shows broad prospects in high-end fruit and vegetable quality control and can be extended to other perishable agricultural products, such as apples and mangoes.

#### 2.3.4. Deep Learning

DL generally includes CNNs [109,110,111], recurrent neural networks (RNNs) [112,113,114], transformer, and automatic encoder (AE). It constructs a multi-layer deep model by simulating a human brain neuron network to automatically learn abstract features from data. DL is used in the non-destructive testing of food, prediction of food chemical composition, real-time monitoring of microorganisms, and rapid identification of pesticide residues in fruits and vegetables. For the monitoring of meat freshness, Gong et al. [115] introduced a smartphone platform based on a gelatin–methylacrylyl (GelMA) hydrogel combined with the DL model. Bromocresol green dye was encapsulated in the GelMA hydrogel through ultraviolet crosslinking technology to make a colorimetric indicator strip that changed color with volatile nitrogen compounds produced by food degradation. The indicator strip image was captured by a smartphone and the classification was carried out by using the dataset. Combined with the application program developed by the CNN and the watershed algorithm, the automatic classification and recognition of meat freshness were determined with 96.2% prediction accuracy. This platform is non-destructive, real time, and portable, providing an intelligent food freshness evaluation solution for the food industry and consumers.

### 2.4. Critical Analysis of AI Technologies in Food Safety

In the field of food safety detection, more AI technologies are integrated with traditional detection technology. This integration gives rapid and accurate information on food quality and contaminants, improves detection efficiency and accuracy, and resolves the shortcomings of traditional detection methods, which are time-consuming and error-prone. In addition, the early risk warning and traceability system based on AI can deeply analyze the data of food source quality control, circulation, storage, production, and sales stages. This AI-integrated system can sense potential risks, accelerating the process of traditional manual monitoring. This system integrates information from all stages of the food supply chain to ensure that when safety problems arise, the source is quickly and accurately detected. This will assist regulatory authorities in efficient disposal and build a comprehensive food safety system from farm to fork.

The integration of AI technology with traditional detection technology has improved detection efficiency and accuracy, but different AI technology branches have their own advantages and disadvantages. Supervised learning has great advantages in the case of explicit classification criteria, but it relies on data labeling. Unsupervised learning can detect unknown risks, but the results are not interpretable enough. Semi-supervised learning can balance efficiency and cost, but the model complexity is high. DL is good at processing high-dimensional data, but it has problems such as high computing power demands and “black box” characteristics. The internet of things (IoT) has significant advantages in real-time dynamic monitoring scenarios, but there are challenges with data heterogeneity and transmission delay. Blockchain has significant advantages in the scenario of building a trusted traceability system, but it suffers from the problems of high storage costs and collaborative governance. The advantage trade-off of AI technology in food safety is essentially a 3D balance of technical characteristics, scenario requirements, and cost constraints. There is no optimal technology, only the most suitable scenario. Each AI technology has its own advantages in accurate classification scenarios, but it needs to break through the limitations of a single algorithm through cross-technology integration. Unsupervised learning and semi-supervised learning are more cost-effective in cost-sensitive scenarios, but they need to cooperate with blockchain and other technologies to improve the credibility of the results. DL and IoT have outstanding advantages in processing complex data and realizing real-time decision-making. However, it is necessary to integrate explainable AI (XAI), edge computing, semi-supervised methods, unsupervised methods, and blockchain technology to build a hybrid intelligent system with complementary advantages and synergy. In the future, as technologies such as edge computing and multimodal models mature, AI will be upgraded from a single-point optimization tool to a full-chain intelligent center to truly realize the technical value of AI in food safety governance and the further upgrade from risk prevention and control to value creation.

In order to understand the technical boundaries and optimization paths of AI technologies in food safety applications, the pros and cons of different algorithms are systematically evaluated in Table 1. Their adaptation scenarios are identified through comparative analysis, which provides a critical perspective for technology selection and cross-domain integration.

## 3. Intelligent Application of AI from Farm to Fork

### 3.1. AI-Based Food Source Management in Farming

For food source safety, disease and pest monitoring, the accurate application of fertilizers and pesticides, the pre-harvest interval (PHI), residue control of fertilizers and pesticides, and the dynamic optimization of the planting environment are the fundamental guarantees of raw food material quality and ingredient safety. Intelligent monitoring, big data analysis, and biological and physical control methods strengthen the prevention and control of pests and diseases [116,117]. Modern agricultural machinery, such as drones and intelligent sprays, achieves accurate pesticide application; strict residue detection systems ensure the safety of agricultural products; and soil health management, water-saving irrigation, and climate-adaptive planting optimize the planting environment [118,119,120]. The new model of AI+ agriculture shows great potential in regulating the safety of food sources.

**Figure 3:**
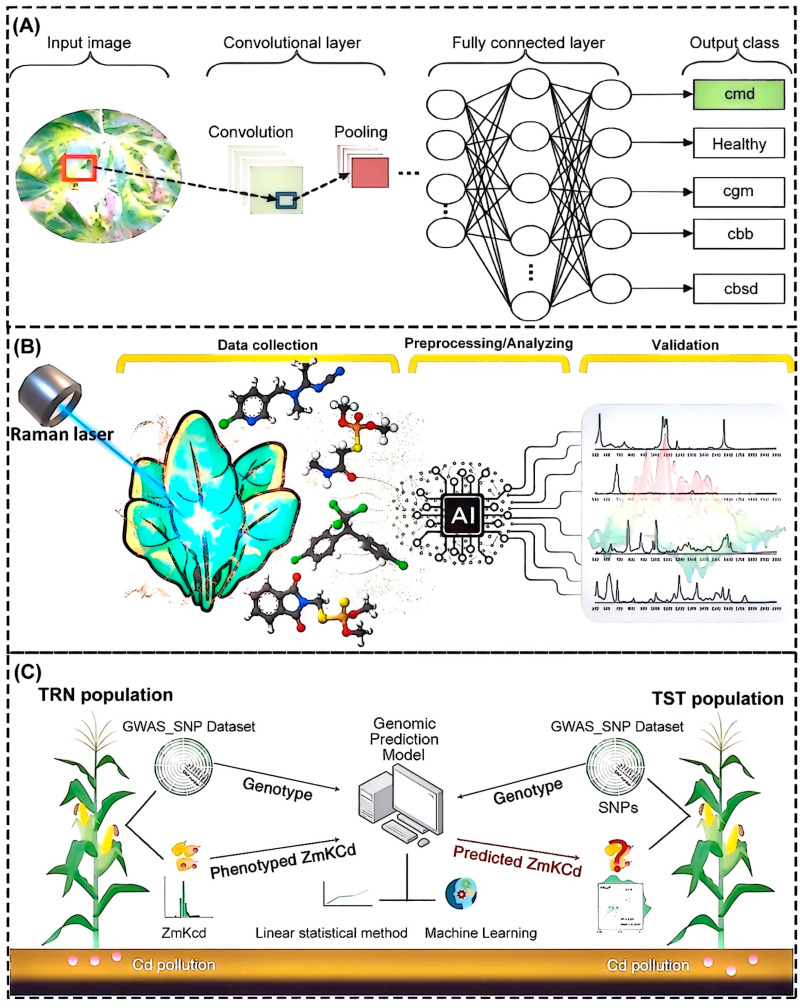
Application of AI in food source quality control. (**A**) CNN model framework for the classification of cassava leaves infected by pests and diseases. (**B**) SERS combined with AI to detect pesticide residues on vegetable surfaces. (**C**) ML algorithm for the detection of heavy metal content in maize grains.

The combination of neural networks and machine vision technology is commonly used to detect surface, sub-surface, and internal defects in fruits and vegetables and monitor plant pests and diseases. Multidimensional plant leaf image data generated by RGB cameras, multi-spectral imaging, hyperspectral imaging, X-ray, and other imaging technologies are often used for the identification of leaf and fruit defects. To identify more complex internal lesions, neural networks are used to automatically learn abstract features in images through multi-layer iteration [121]. Compared with traditional machine vision, which relies on artificial features to extract color, shape, and texture characteristics, the detection accuracy is higher [122]. Sambasivam et al. [123] trained a CNN for the disease detection and classification of cassava leaves in the agricultural sector based on field datasets collected in Uganda from the Kaggle competition. The complete classification process is represented graphically in Figure 3A. The dataset contained 10,000 labeled images across five categories, with healthy leaves making up only 3.16%, indicating a significant category imbalance. By building a custom CNN model with three convolution layers and four fully connected layers, combined with data enhancement and SMOTE oversampling, the class bias was effectively reduced. Hyperparameters were adjusted by grid search and three-fold cross-validation and, finally, 93% accuracy was achieved under the input resolution of 448 × 448 pixels. This proved the robustness of the CNN under the condition of limited and unbalanced data, providing a feasible scheme for real-time disease diagnosis in resource-limited areas. In order to make it easier for multiple AI technologies to monitor plant pests and diseases, Christakakis et al. [124] developed a cross-platform mobile application that could identify damage in tomato crops caused by tomato leaf miners by using the real-time detection of plant diseases and pests through AI and DL technology. An infected crop image was transferred to the back-end system through REST API and the disease features in the image were analyzed in real time by the pre-trained YOLOv8 model. After processing by the support system and the compilation of detection results, including disease location, confidence, and management suggestions, YOLOv8 ensured robustness for real-time inspection on mobile devices in complex field environments with 87% accuracy and directly reduced manual inspection errors.

The precise application of chemical fertilizers and the monitoring of pesticide residues are crucial for food safety [125,126,127]. The illegitimate use of pesticides to protect crops can lead to residues in final products; these poisons are not only directly related to the quality and safety of food but also affect the sustainable development of agricultural production and the protection of the ecological environment [128,129]. The precise application of these chemicals can effectively reduce residues. Hajikhani et al. [130] developed a novel detection method combining surface-enhanced Raman spectroscopy (SERS) and a transformer model for detecting pesticide residues in spinach (Figure 3B). The model processed a labeled SERS dataset with a shared-weight transformer encoder layer, which contained specific pesticide types and pesticide labels. The classification branch used the six-category Soft Max classifier to identify pesticide types, and the regression analysis predicted concentrations through the machine language program. This method could be used for qualitative and quantitative analysis with accuracy of 98.4%, mean absolute error (MAE) of 0.966, and mean square error (MSE) of 1.826, showing good sensitivity and selectivity. SERS was also used for the detection of pesticide residues on the surface of fruits. Wang et al. [131] combined SERS and CNN innovative methods for the qualitative and quantitative detection of pesticide residues on peel surface. The CNN model automatically extracted spectral features without manual selection, which effectively solved the problem of SERS data complexity. The mapping relationship between spectra and pesticides was established through the training set to achieve the accurate classification and detection of a variety of pesticides. A prediction accuracy of 99.62% was achieved on the test set, which was significantly better than ML models such as SVM and RF. This method provided an efficient solution for the rapid screening of multiple pesticide residues.

In addition to organic pollutants and pesticide residues, heavy metal ions such as lead, cadmium, mercury, and arsenic also pose a serious threat to food safety. These heavy metal ions can accumulate in the body, resulting in chronic poisoning and damage to the nervous system, hematopoietic organs, kidneys, and other systems [132,133,134,135]. For example, lead can affect the growth and development of children, cadmium can cause “Itai-itai disease”, and mercury can cause “Minamata disease”, so it is crucial to ensure that heavy metal levels in food are within acceptable limits. Heavy metal enters our food chain either from the growing environment or through raw material processing [136,137,138]. In order to detect these heavy metal ions in food, Mandal et al. [139] developed a detection method based on a combination of a carbon nanoparticle fluorescence sensor array and DL. Nine different surface-functionalized fluorescent carbon nanoparticles were synthesized by the hydrothermal method to build a sensor array. These carbon nanoparticles generated distinguishable visual features under ultraviolet excitation when they interacted with heavy metal ions. Fluorescence images were captured by digital cameras, RGB values were extracted as feature data, and the original 86 data points were enhanced by generating adversarial networks to form an enhanced multi-layer perceptron model. Finally, ML algorithms such as the multi-layer perceptron were used for the high-precision classification and recognition of five heavy metal ions, achieving 83.1% accuracy in three-fold cross-validation. This detection method was significantly better than the traditional methods. By combining genome-wide association analysis and genomic prediction techniques, Yan et al. [140] developed a hybrid model based on ML and linear statistics methods to assess genotypic–phenotypic relationships for cadmium concentration in maize grains. The research focused on screening single nucleotide polymorphism markers associated with cadmium accumulation and optimizing model parameters and compared the prediction performance of the Bayesian method, ridge regression, the best linear unbiased prediction (rrBLUP) and RF algorithms, as shown in Figure 3C. The results showed that the rrBLUP had the highest prediction accuracy in field trials. The mean correlation coefficient was 0.89 and the MAE was 0.0037, which provided a biological basis for revealing the molecular mechanism of cadmium accumulation and provided data-driven decision support for precision agriculture and food pollution prevention and control.

**Figure 4:**
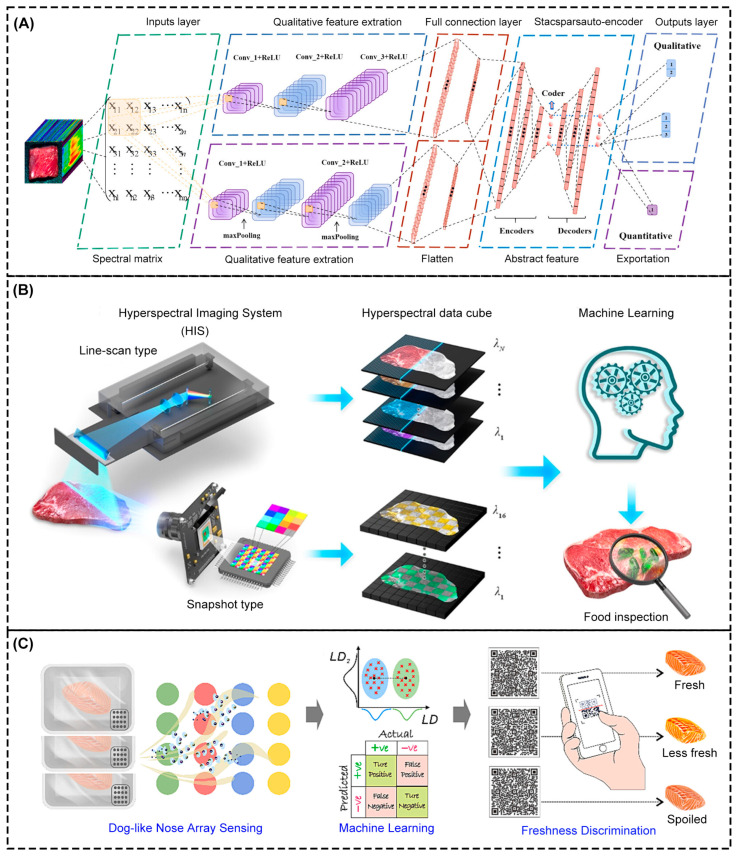
Application of AI in meat freshness assessment. (**A**) Qualitative and quantitative testing of mutton using CNN-SSAE model. (**B**) Schematics of the full process required to obtain hyperspectral data and evaluation using ML for food inspection. Two hyperspectral imaging systems were used for hyperspectral data acquisition: a line-scan-type HIS installed with a commercial grating for high spatial and spectral resolution and a snapshot-type HIS for compact form factor and efficient computation resources. ML was conducted to extract FI values from the hyperspectral data for food inspection [147]. (**C**) Freshness prediction and real-time detection scheme of array gas sensor based on the LDA model.

In animal husbandry, in order to prevent and treat animal diseases, various veterinary drugs are often used [141]. However, if animals are slaughtered during the drug action interval, drugs or their metabolites may accumulate and concentrate in animal tissues. The long-term intake of food contaminated with veterinary drug residues may lead to health problems such as decreased human immunity and endocrine disorders. Therefore, it is crucial to monitor these veterinary drugs to ensure that the food is safe for human consumption [142]. Dong et al. [143] proposed a rapid detection method for ofloxacin (OFX) veterinary drug residues in mutton based on hyperspectral imaging technology combined with XAI, as shown in Figure 4A. By collecting near-infrared hyperspectral data of 300 groups of mutton samples with different OFX residue concentrations, a convolutional neural network-stacked sparse auto-encoder model was constructed for the qualitative and quantitative detection of OFX residues. The CNN, as the core architecture, enhanced the feature extraction ability of small sample data and achieved 93.65% prediction accuracy. Compared with the traditional chromatographic detection method, this method has the advantages of conducting non-destructive detection and real-time analysis and decision-making processes for the detection of antibiotic residues in meat.

The detection of chemical fertilizer, heavy metals, and pesticide and veterinary drug residues in food commodities ensures safety. Adjusting crop planting structure and improving other agricultural management practices can significantly improve the quality of food. This optimization not only reduces the input of toxic substances but also reduces their detrimental effects on the environment. Zhu et al. [144] developed a web-based CPDOS AI platform to increase crop yields by optimizing crop planting density and fertilization strategies. The system integrates AI technologies such as genetic algorithms and backpropagation neural networks and combines multiple yield density models to develop core modules, including yield density, optimal planting density range, and joint optimization modules of fertilization and planting density. The effectiveness of the system was validated on potato, corn, and soybean. The average determination coefficient of the genetic algorithm in the three crop density models reached 98.18%. The system integrates algorithms and web technology, realizes data visualization and automation processes through B/S architecture, lowers the threshold for agricultural practitioners to use complex models, and promotes the actual landing of intelligent planting technology. While optimizing planting technology, environmental factors such as temperature and climate also affect the quality and yield of crops. Decardi et al. [145] used AI to optimize the energy efficiency and resource management of plant nurseries with artificial lighting (PFALs) and automatically adjusted artificial lighting and climate systems in plant factories. By building a model of plant–environment interaction, the deep reinforcement learning agent selected the optimal control actions that reduced energy consumption by 25% and improved crop yield. The results showed that a soft actor–critic algorithm could reduce the energy consumption of PFALs from 9.5–10.5 kwh kg^−1^ to 6.42–7.26 kwh kg^−1^ by dynamically adjusting the lighting and climate control system and revealed the tradeoff between energy saving and CO_2_ utilization in ventilation. By optimizing the light and climate system and enabling personalized regulation according to plant species and growth stage, this AI technology can directly increase the yield and quality of agricultural products, enhance sustainability and food safety, and ultimately improve the supply capacity and quality level of raw food materials.

### 3.2. AI-Based Sorting in Food Ingredients

Intelligent sorting before preprocessing can quickly and accurately classify raw food materials and eliminate low-grade products before the preprocessing stage. The fusion of multi-spectral data and ML algorithms improves sorting efficiency, reduces the risk of food contamination, and provides more reliable food products. Magnus et al. [146] proposed a novel cascade classifier architecture to solve the problem of sample variability in food detection by traditional stoichiometric methods. The architecture integrated the data of reflection and fluorescence spectra, adopted a feature selection strategy to reduce detection wavelengths to 8, and integrated models such as extreme learning machines and SVMs. Through the mapping relationship between spectral data features and known class labels, the classifier could effectively distinguish normal and defective walnuts, and the correct classification rate of various defective samples was more than 98%. So, it was concluded that the fusion of multi-spectral data and the ML algorithm showed high accuracy in food sorting. These techniques also demonstrate great application potential in evaluating meat freshness. Lee et al. [147] used hyperspectral imaging to capture the morphological and fluorescence spectral information of meat surfaces, defined a freshness index based on the ratio of reduced nicotinamide adenine dinucleotide and myoglobin fluorescence intensity, and correlated it with bacterial density, as shown in Figure 4B. Two device methods, line scanning and snapshot, were used in the study, combined with ML algorithms such as linear discriminant analysis (LDA) and quadratic discriminant analysis. Features were extracted from hyperspectral data and classification models were established. Finally, the system was integrated with a smartphone to detect meat freshness in real-time and remotely. The FI value based on the fluorescence spectrum showed a linear relationship with bacterial density (R^2^ = 0.99). Qu et al. [148] developed a novel gas array sensor based on SERS technology, which was used for the multidimensional detection of volatile organic compounds (VOCs) in food and combined with an ML algorithm to achieve the real-time assessment of food freshness, as shown in Figure 4C. The plasma array substrate prepared by the interface self-assembly method selectively captured four VOCs using a metal–organic framework and different monolayer layers. The sensor obtained multidimensional spectral fingerprint information by combining direct and indirect SERS signals. Combined with PCA dimensionality reduction and the LDA training model, a complete analysis process was formed. Experiments showed that the four-dimensional LDA model had a classification accuracy of 96.9% for fresh, sub-fresh, spoiled, and rotten samples. This method removes the limitations of traditional SERS single-target detection and gives high-accuracy and low-interference analyses of complex VOC mixed systems. For the authenticity detection of meat food, the fusion of multi-spectral data and the ML algorithm is still applicable. Parastar et al. [149] developed a fast, non-destructive method to detect the authenticity of chicken meat by combining handheld near-infrared spectroscopy and ML algorithms. In this study, different measurement modes were used to classify chicken meat. By obtaining spectral data through the top and bottom of the package, fresh and thawed chicken were classified through a random subspace discriminant integration algorithm. The classification was quick and, with more than 95% accuracy, rapid detection through the package could be obtained. The complex spectral patterns were resolved by the ML model, which removed the obstacles of traditional chemical analysis methods.

### 3.3. AI-Based Food Storage Monitoring in Warehouses

After the strict control of the quality of source crops and the sorting of defective food before pre-processing, an intelligent prediction system of food storage safety should include the last shift of food into the production line. Through the integration of advanced means such as the internet of things +AI technology, an intelligent prediction system can monitor the storage environment in real time and accurately analyze the risk of food deterioration. This effectively extends the shelf life of food and ensures the freshness and safety of food. For grain storage, Wang et al. [150] used grain quality indicators, temperature, and humidity to construct predictive models to dynamically analyze grain quality changes during storage. Two models were proposed in the research, namely, the intertemporal prediction model based on BP neural networks and the synchronous prediction model based on SVMs. These models are applicable to wheat, corn, rice, and soybeans. Combined with the stored data of different ecological regions, the prediction errors of the two models were controlled within 15% to 20%. Both models could effectively predict key storage characteristics with an error of 15–20%. Westerveld et al. [151] developed a portable food storage security prediction model by integrating multi- and open-source data with the XGBoost algorithm. Taking the living area as the spatial unit, the study defined the change in food safety status based on comprehensive stage classification data and collected a total of 130 open-source datasets in 19 categories as predictors. Lagged variables and seasonal indicators were generated by feature engineering and the ADASYN algorithm was used to deal with class imbalance. The results indicated that the model performed better in the 7-month forecast horizon compared to the 3-month short-term forecast, achieving an F1 macro average of 0.61 for July and an F1 macro average of 0.51 for March. For disease and insect attacks on stored food, Wu et al. [152] used DL technology to automatically identify common beetle species, and a dataset was built by collecting micrograph images of elytra fragments from 15 storage beetles. The CNN model based on the VGG16 architecture and the transfer learning method was used to classify beetle species. At the same time, to overcome the challenge of the limited sample size, 6900 elytra fragment images were used, finally achieving an overall classification accuracy of 83.8% in cross-validation. This provided a scalable and intelligent analysis tool for food storage. For the shelf-life prediction of meat at different temperatures, Cui et al. [153] developed a multi-objective real-time prediction model using an ML algorithm to simultaneously predict the shelf life of five fish products at different storage temperatures by monitoring 14 characteristic indicators such as total live count, volatile base nitrogen, K value, electronic nose, gas chromatography–mass spectrometry data, and sensory evaluation data. This model combined four ML models: the BP neural network, BP neural network optimized by the genetic algorithm, radial basis function (RBF) neural network, and extreme learning machine. A shelf-life prediction model of marine fish under multi-species and multi-temperature conditions was established. The results showed that the RBF model had the smallest prediction error: the absolute error was less than 0.5 days, MAE = 0.118, and R^2^ = 0.9994. Based on the RBF model, a real-time prediction platform was developed, which provides technical support for quality monitoring in the food supply chain and intelligent solutions for reducing food losses.

In order to ensure food quality and safety, advanced sensor technology should be used to monitor key indicators such as temperature and humidity, gas composition, and microbial activity. In cases of abnormalities, storage conditions should be adjusted to reduce food loss. In the interim, big data analysis should be used to predict the shelf life of food. Precise management should be undertaken for perishable and difficult-to-store foods during storage to protect the health and safety of consumers. Formalin is mostly used to preserve fish by controlling parasitic infection in fish skin. Mahata et al. [154] used a SnO_2_ nano-structured gas sensor combined with ML technology to detect formalin residues in fish. A dataset containing the dynamic response characteristics of the sensor was constructed by comparing the VOC differences of fresh tilapia under different handling and storage conditions. RF and SVM achieved a theoretical detection limit for formaldehyde as low as 75 ppb by constructing a decision boundary to classify the three samples with 95.83% accuracy. Similarly, a DNN model accurately predicted food storage time by establishing nonlinear mapping between response amplitude and storage time, and the regression average error was less than 8.28%. This method combined the high sensitivity of a physical sensor with the advantage of ML pattern recognition to enable the intelligent conversion from signal detection to quality assessment. This method also provides an extensible technical framework for the safety monitoring of perishable and difficult-to-store foods. The applications of AI in quality control at the source of food, intelligent sorting before pre-processing, and food storage safety are summarized in Table 2.

### 3.4. AI-Based Quality Control in Food Processing

**Figure 5:**
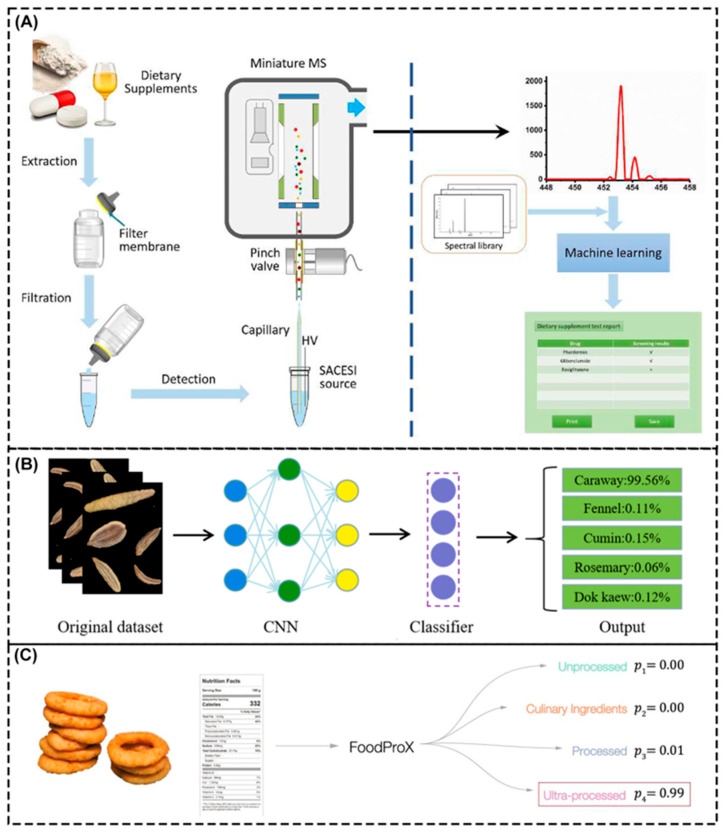
Application of AI in the intelligent control of food processing. (**A**) Schematic diagram of AI combined with micro-mass spectrometry for detecting food adulteration. (**B**) Spice classification flow chart based on an improved CNN model. (**C**) Classification flow chart of FoodProX for predicting the degree of food processing.

The introduction of AI technology, such as DL and machine vision, into the food processing chain can achieve more efficient and accurate quality control. These technologies can monitor various parameters on the production line in real time to ensure that the processing conditions meet food safety standards. For the detection of adulteration, Shi et al. [155] developed a self-priming capillary electro spray ionization source and a two-step pretreatment method, combined with the ion trap analyzer and ML algorithm, to achieve the efficient detection of illegal food additives without chromatographic separation, as shown in Figure 5A. A mass spectrum library containing 31 illegal substances was contracted including, for instance, Sudan Red, borax, and melamine. A SVM model was trained to classify and identify unknown samples using functional food containing illegal substances as positive samples and unadulterated matrices as negative samples. The model could complete sample pretreatment and detection within 1 min and was successfully applied to the field screening of 55 batches, with an accuracy rate of 100%, which significantly improved the detection efficiency and portability. In terms of adulteration detection in food processing, Ni et al. [156] developed a condiment identification method based on an improved CNN for the classification of five condiments with similar appearance but different effects to solve the problem of food adulteration, as shown in Figure 5B. Based on the residual network 18 (ResNet18) model, three improvements were made in the study. Spatial and channel squeeze and excitation were introduced, the convolution kernel size of the last residual module was adjusted, and the classifier structure was optimized. A recognition accuracy of 95.71% was finally achieved, which was 1.11% higher than that of the original ResNet18. This approach overcomes the dependence of traditional methods on professional equipment and complex operations, such as near-infrared spectroscopy and chemometrics. This approach enables low-cost and high-efficiency detection based on conventional images and provides a new technical path for intelligent food detection.

The degree of food processing is significantly associated with physical health, and the higher the degree of processing, the greater the potential harm to health. Ultra-processed foods are significantly associated with the risk of metabolic syndrome, diabetes, and other diseases [157]. By using alternative strategies, it is found that replacing a small amount of ultra-processed foods can significantly improve health indicators. For predicting the degree of food processing, Menichetti et al. [158] proposed FoodProX, an ML-based classification model for predicting the concentration changes of food nutrients, as shown in Figure 5C. In the study, FoodProX standardized nutrient composition data from the USDA database as the input of the RF and output the distribution probabilities of unprocessed and processed raw materials and processed and ultra-processed foods. Through the cross-verification of 50 folds, the model showed high differentiation in the mentioned four classes. The area under the curve was more than 0.96. Finally, the continuous processing score index was generated by probability weighting to quantify the gradient of the processing degree. In terms of the regulation of harmful ingredients in food packaging, Wang et al. [159] built an improved radial basis function artificial neural network (RBF ANN) model based on gas chromatography–mass spectrometry experimental data to predict phthalic acid under different temperature, time, and food analog types. At the same time, a molecular dynamics simulation was used to analyze the interaction, solubility parameters, and free volume fraction of polyvinylidene chloride packaging materials with food simulants. The results showed that the model had a high correlation coefficient of 0.95, low prediction error, and MSE of 0.046, accurately predicting migration behavior in complex environments. Moreover, it was found that temperature was the key factor affecting the migration, which provides a multi-scale analysis framework for the migration mechanism of toxic components in packaging materials.

### 3.5. AI-Based Detection in Food Products

In food safety testing, the combination of AI and rapid detection technology has brought revolutionary changes to the detection of microorganisms, spoilage, and toxicity [160,161]. Using AI algorithms, rapid detection technology can identify harmful substances in food more accurately and efficiently, realize on-site detection, reduce the detection cycle, and improve the practicality and timeliness of food safety monitoring [162,163,164].

**Figure 6:**
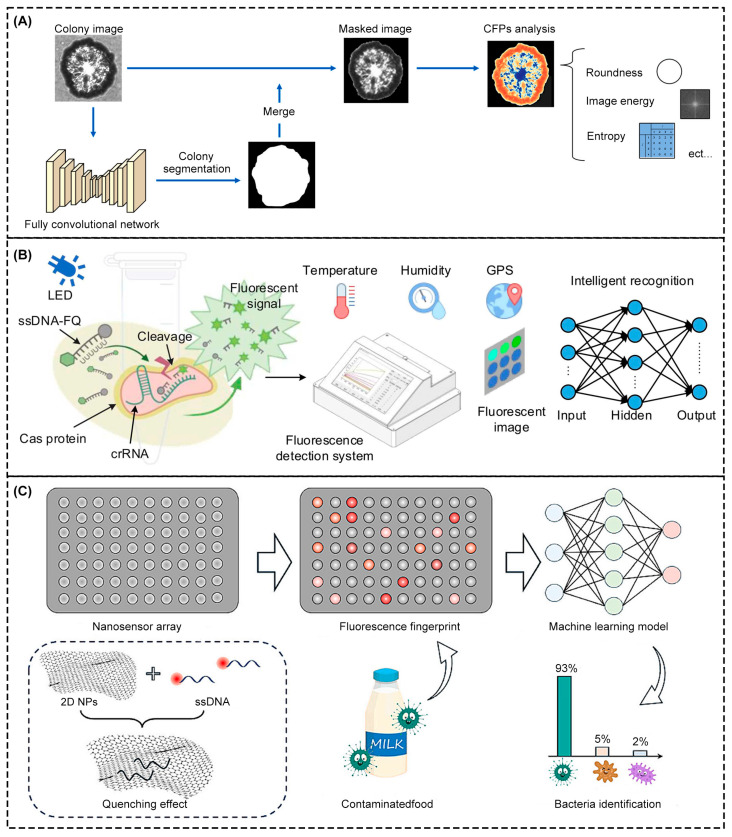
Application of AI in the rapid detection of foodborne pathogens. (**A**) Image processing steps for colony fingerprint analysis from clipped colony images. (**B**) The working principle of a portable, optical fiber, real-time fluorescence detection system based on radial basis function neural network algorithms for detecting pathogenic microorganisms in food. (**C**) ANN model-based fluorescent sensor arrays for identifying multiple foodborne pathogens and spoilage bacteria in milk.

For the rapid detection of foodborne pathogens, Tago et al. [165] developed a new imaging system of bacterial colony fingerprints based on a line image sensor. Wide-field imaging of bacterial microcolonies using high-speed wire sensors could scan an entire 92-mm diameter petri dish in 22 s. By extracting morphological, optical, and textural features during colony growth and constructing a classification model with the XGBoost algorithm, the high precision and rapid identification of 15 species of bacteria were achieved (Figure 6A). This method could detect *Staphylococcus aureus* in 10 h with 96% accuracy in food samples, which significantly reduced the detection cycle compared with the traditional 24-h mass spectrometry method. Guo et al. [166] introduced a new, portable, optical fiber, real-time fluorescence detection system for the high-precision field detection of pathogenic microorganisms in food (Figure 6B). By integrating an LED light source, optical fiber sensing array, and micro-camera, the system enabled multichannel fluorescence signal acquisition. It also combined environmental parameter sensing functions such as temperature, humidity, and GPS positioning and innovatively combined a radial basis function neural network algorithm with digital signal processing technology to solve the problem whereby traditional detection methods are susceptible to environmental interference. The model processed multidimensional input data through a hidden layer with a Gaussian kernel function and output the predicted concentration of target microorganisms. The model showed high sensitivity and accuracy for the detection of African swine cholera virus and Salmonella, with detection limits of 2.5 CFU/μL and 10 CFU/mL, respectively. The model supported dynamic monitoring during transportation. Wang et al. [167] developed a fluorescence sensor array based on single-stranded DNA and two-dimensional nanomaterials, combined with an ML algorithm, for the rapid detection and identification of multiple foodborne pathogens and spoilage bacteria in milk (Figure 6C). The sensor array could generate unique fluorescence recovery fingerprints for different bacteria; after standardization, the algorithm extracted characteristic patterns from the fluorescence recovery intensity of 12 sensor units. The mapping relationship between bacterial species and fluorescence response was established, the complex multidimensional data generated by non-specific sensors was effectively analyzed, and the limitation of traditional single biomarker detection was overcome. Moreover, the accuracy rate of the ANN model reached 93.8% under the short-term incubation condition of 30 min, showing the advantage of the ANN in handling nonlinear relations. This method realizes the simultaneous identification of multiple bacteria through a multi-sensor cooperative response and algorithm optimization and has better detection throughput and cost-effectiveness than traditional methods, which provides a new strategy for the rapid microbial detection of food substrates.

**Figure 7:**
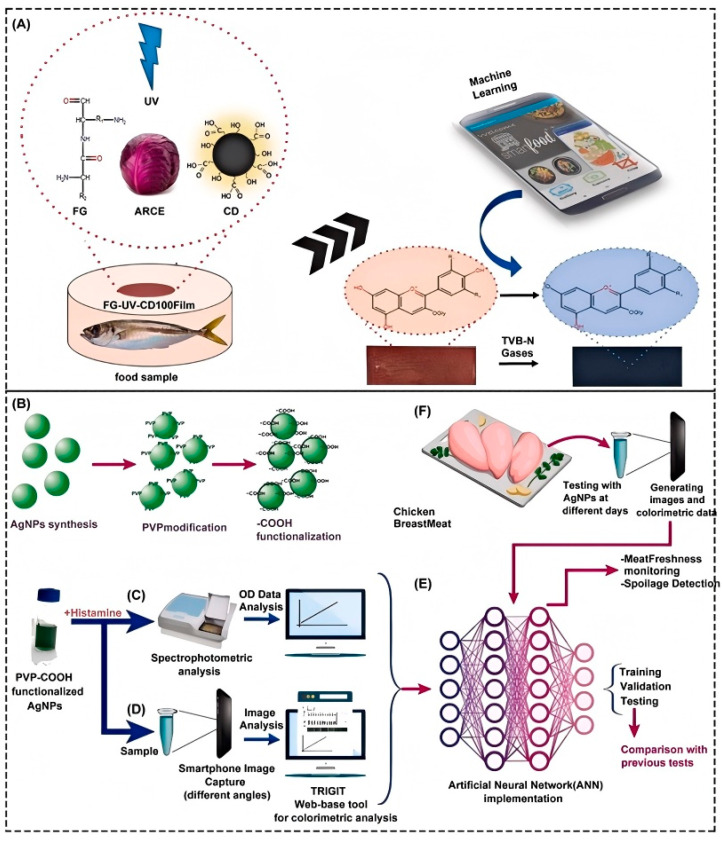
Study design for the rapid assessment of meat freshness based on the colorimetric method. (**A**) Fish freshness detection based on portable colorimetry. (**B**) Schematic diagram of synthesized silver nanoparticles. (**C**) Histamine spectrophotometric analysis. (**D**) Histamine analysis based on smartphone images. (**E**) Using colorimetric data for ANN model training. (**F**) Simulation of a real chicken sample contrast color sensing system for testing.

In food safety, an intelligent detection system can complete rapid and on-site detection of food spoilage through a combination of the colorimetric method and the ML algorithm [168]. Dogan et al. [169] built a smartphone-embedded, ML, on-site, colorimetric food spoilage monitoring system. They developed a fish gelatin film adulterated with red cabbage extraction that showed color changes when exposed to volatile amines produced by food spoilage. By integrating ML algorithms and smartphone applications, the rapid classification and recognition of ammonia concentration gradient responses were achieved, as shown in Figure 7A. After selecting and optimizing features based on the Chi-square test, the RF classifier was adopted to achieve 98.8% accuracy, and the training model was innovatively embedded in Android Smartfood ++. The offline detection of food spoilage in real fish samples was achieved in 0.1 s with 99.6% accuracy. These improvements remove the limitations of traditional colorimetric methods that rely on manual interpretation and cloud computing. Ghorbanizamani [170] developed a colorimetric sensing system by combining silver nanoparticles, smartphone imaging, and ANN models to detect bioamines produced during chicken spoilage, as shown in Figure 7B–F. Through the interaction of green, synthesized silver nanoparticles with biogenic amines, the color changes were triggered, and the RGB color parameters were captured by a smartphone. By optimizing the reaction conditions, the sensor detected the initial biogenic amines with a detection limit of 0.21 μg/mL. In order to further improve the performance, the researchers combined various color parameters extracted from smartphones with spectral data to train the ANN model and finally improved the detection limit to 0.09 μg/mL and extended the dynamic range to 0.5–200 μg/mL, and the R^2^ of the ANN model was 0.9946. The effectiveness of the system in three days’ spoilage monitoring was verified by actual chicken samples, which proved that the system could reflect the degree of spoilage.

### 3.6. AI-Based Blockchain for Food Traceability

**Figure 8:**
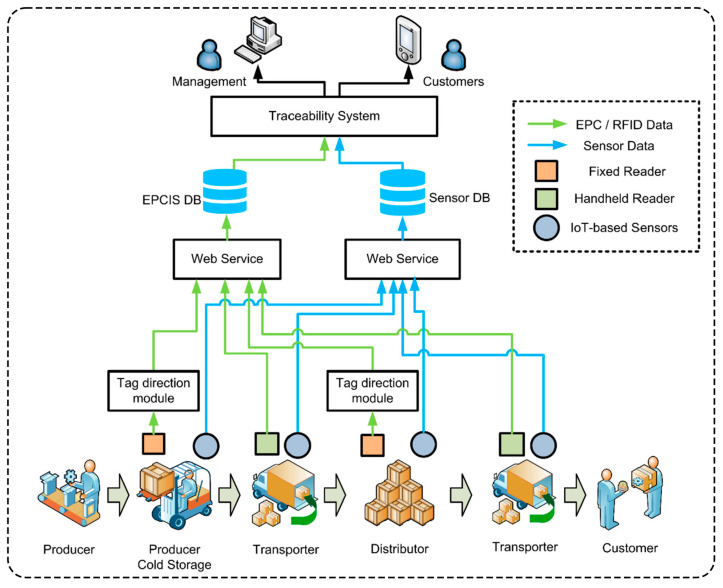
Trusted traceability architecture with blockchain +AI, where food moves from producers to consumers through multiple supply chains such as distributors and transporters.

The most important feature of the trustworthy traceability architecture of blockchain +AI is immutability [171,172,173,174]. Using blockchain technology and AI’s intelligent analysis ability, the information of key stages such as the source, production and transportation of each batch of raw materials is accurately recorded and immutable, thus forming a complete traceability chain, effectively preventing the inflow of counterfeit and substandard products while enhancing the credibility and security of food [175,176,177]. It provides a solid guarantee of food safety. Richter et al. [178] proposed a method for the rapid screening of the geographical origin of white asparagus using near-infrared spectroscopy combined with an SVM model. After the asparagus samples were freeze-dried and ground, Fourier transform near-infrared spectral data were obtained, an SVM was used to establish a classification model, and the feature selection and confidence estimation methods were combined to distinguish the country of origin of the asparagus. The accuracy of differentiation between German asparagus and other countries reached 89%. The study proved the practical value of near-infrared spectroscopy combined with ML in the field of food traceability and provided an efficient technical means for the protection of geographical indications of agricultural products and food origin traceability. Li et al. [179] used two-dimensional gas chromatography–time-of-flight mass spectrometry to obtain complex composition data of 262 Chinese liquors of different geographical sources and flavor types. The chemical characteristics of different flavor types were revealed through PCA and hierarchical clustering, and, for further analysis, SVM and RF ML models were used. The high precision classification of Chinese liquor was successfully obtained with accuracies of 91.86%, 97.67%, 83.72%, and 95.36%, respectively. Alfian et al. [180] developed a traceability system based on radio frequency identification, combined with internet of things sensors to monitor real-time temperature and humidity data during storage and transportation. The XGBoost algorithm was used to solve the problem of radio frequency identification label direction recognition, as shown in Figure 8. Through experimental verification, the XGBoost model performed well in the test, with an accuracy rate of 93.59%, a recall rate of 92.95%, and an F1 score of 92.78%. The system could effectively distinguish between label entry and exit directions, such as receiving or shipping, while providing complete supply chain history and environmental monitoring data, thereby improving the efficiency of food quality and safety management.

Blockchain +AI technology not only strengthens the transparency of the supply chain but also enhances the standards of accountability in the food industry. At the same time, AI also demonstrates a strong ability to predict defective product categories, hazard types, and disposal measures. By analyzing large amounts of food testing data, consumer feedback, and production process information, AI can identify potential safety hazards in different foods and accurately predict the product categories that may have problems. Whether this involves microbial pollution, excessive additives, or deterioration due to expiry, AI can quickly make judgments and provide timely warnings for regulatory authorities and enterprises. Nogales et al. [181] introduced the application of ML models in predicting food and feed safety risks. By comparing the performance of neural and non-neural models in three food safety prediction tasks, the impact of the classification variable coding strategy on model performance was analyzed. It was found that the neural network model combined with entity embedding coding achieved the highest accuracy in predicting the product category, hazard category, and treatment measures, with accuracies of 86.81%, 82.31%, and 88.94%, respectively. This approach integrates structured classification data and DL models through a data-driven risk warning system to improve the initiative and resource utilization of food safety supervision.

### 3.7. AI-Based Personalized Meal Services for the Table

In the field of food safety and health, a personalized, intelligent nutrition service management system for the whole life cycle can be achieved by combining the customization of food sensory characteristics and nutrient matching, dynamic food demand prediction based on individual characteristics, and the prediction of the influence of food components on disease-related protein pathways [182]. Mengucci et al. [183] explored the key role of food structure in sensory properties, stability, and nutrient digestion and absorption and proposed a predictive model framework using AI technology to build food structure and function. The description of traditional food is mostly based on chemical composition analysis, but many important characteristics are determined by the micro and macrostructure. The research systematically reviewed the definition of food structure, measurement methods, and the mechanism of its influence on function. Taking the structural changes in the cooking process of pasta as an example, a modeling process integrating multi-scale structural data and kernel principal component analysis (KPCA) was proposed. The aim of this approach was to reduce the dependence on physical experiments through digital twin technology, enabling the computer-aided design of food attributes and providing a computing platform for personalized food design and a new path for precise nutrition collocation and personalized food development.

Dynamic food demand forecasting based on individual characteristics is an innovative tool integrating intelligence and environmental protection concepts. Through big data analysis of users’ eating habits and preferences, it accurately predicts future food demand, enables the accurate management of food procurement and consumption, automatically balances meals according to actual needs, ensures balanced nutrition, effectively reduces food waste, and promotes resource conservation. Rodrigues et al. [184] used four ML models—RF, light gradient boosting machine, long short-term memory, and transformer—to predict the next day’s catering demand. The results showed that RF and the long short-term memory model could effectively reduce food waste by 14–52%, and reduce unmet demand by 3–16%. The study also highlighted the importance of accurate demand forecasting in optimizing production plans and reducing resource waste, providing data-driven solutions and actionable decision support for addressing sustainable development issues in the catering industry.

**Figure 9:**
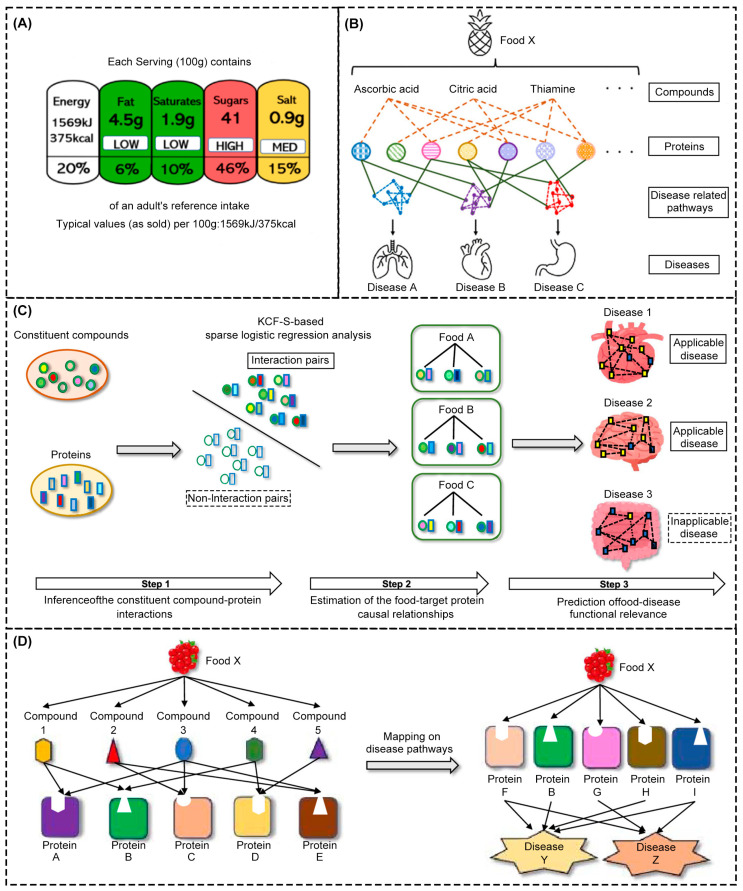
Application of AI in personalized intelligent service systems. (**A**) An AI model was established to rapidly predict the nutrient composition of trace elements in food. (**B**) AI-based methods for predicting functional associations between food and disease. (**C**) The proposed approach consisted of three main steps. In the first step, sparse logistic regression analysis combined with the KCF-S descriptor analyzed the interactions between the component compounds in the food and the target proteins to reveal the molecular interaction mechanism within each food. In the second step, the compound protein interactions derived from the first step were grouped by food to evaluate the causal relationship between the food and the target protein. In the third step, the target proteins of each food were mapped to disease-associated pathways, and pathway enrichment analysis was used to predict which diseases each food may be suitable for [185]. (**D**) The functional association between food and disease was established through the following two steps. First, compound protein interactions derived from machine learning were combined with food composition information to estimate the causal relationship between food and protein. Secondly, the target proteins of the food and disease pathway were analyzed to predict the functional association between food and disease [185].

The prediction of the effect of food composition on disease-related protein pathways is mainly reflected in the balance of nutrients and the activity of specific ingredients. A balanced diet can provide all needed nutrients, boost immunity, and prevent diseases caused by nutrient deficiencies. In addition, the active ingredients in some foods, such as antioxidants, dietary fiber, and probiotics, can directly act on the bodily systems, reduce inflammation, regulate intestinal flora, and reduce the risk of chronic diseases. Through scientific and reasonable food selection, we can not only satisfy the taste buds but also effectively prevent diseases and improve quality of life. Inoue et al. [185] built a prediction framework from food composition to disease pathway by integrating data from multiple sources such as food composition, compound protein interaction, and the influence on disease-related pathways. The L1 regularized logistic regression model was used to assess the interaction between food composition and target proteins, and hypergeometric tests were used to assess the enrichment degree of food target proteins in disease pathways. This was to predict the functional association between food and disease, as shown in Figure 9B–D. The model performed well in the five-fold cross-validation, predicting a mean AUC of 0.92 for compound protein interactions with an accuracy of 84%. The method covered 876 foods and 83 diseases, revealed the potential mechanism by which food regulates molecular pathways through multi-component synergies, and explored food combinations that may have synergistic effects, providing a new computational tool for preventive medicine. Razavi et al. [186], based on 14 basic nutritional indicators such as the calories, fat, and protein of 5624 foods in the Food and Nutrient Database of Dietary Research, constructed models such as random forest and a support vector machine to predict the content of vitamins A, B, C, E, and K and 15 unlabeled micronutrients such as magnesium and zinc, and these prediction models were integrated through the development of mobile applications to help consumers have a more comprehensive understanding of the nutritional composition of food and assist dietary decisions to deal with the global micronutrient deficiency, as shown in Figure 9A. This data-driven approach breaks through the physical space limitations of traditional labels, provides a scalable technology path for mobile health applications, and realizes the transformation of laboratory-level detection capabilities to consumer applications. In Table 3, the applications of AI in the intelligent control of food processing, rapid detection, trusted traceability of blockchain +AI, and personalized intelligent services are summarized.

## 4. Challenges and Future Directions of AI in Food Safety

Under the wave of digitalization, AI technology is profoundly reconstructing the food safety governance paradigm and building a new system of healthy food quality control through intelligent monitoring, prediction, and decision-making, covering the whole chain from farm to fork [187,188,189]. From the intelligent analysis of environmental parameters and the early warning of diseases and pests at the production end to the ingredient detection and specification monitoring in the processing chain, the cold chain logistics tracking in the circulation chain, and nutrition adaptation suggestions at the consumption end, AI technology significantly improves the accuracy and efficiency of food safety management with multi-modal data fusion, real-time decision support, and cross-domain collaboration capabilities. Its technical potential has been verified in pesticide residue detection, microbial contamination, early warning signalling, food adulteration identification, and food storage [51].

Despite the powerful effect of AI and its deepening application in the field of food safety, it still faces many structural challenges, of which the first is data governance [190,191]. Agricultural production data are mostly fragmented and non-standardized and are significantly different due to the environmental parameters, crop variety characteristics, and processing technology in different regions. So, these variations make it difficult to unify data collection standards and the generalization ability of cross-regional models. [192,193]. The second is the contradiction between algorithm interpretability and real-time monitoring. Food detection scenarios require millisecond-level decision-making, but the “black box” feature of the DL model does not meet the requirements of regulatory transparency [194,195]. Moreover, the technology cost is high. Small and medium-sized enterprises face financial barriers to intelligent transformation. The cost of a set of AI quality inspection equipment is usually 5–8 times more than that of traditional equipment, and the budget of small and medium-sized enterprises that account for the main body of the industry is seriously mismatched. In addition, multi-source data fusion technology is not mature, and the integration and analysis ability of heterogeneous data such as biosensor data, supply chain logs, and consumer feedback is insufficient, which restricts the construction of whole-chain risk prediction models [196,197,198]. In Table 4, the challenges and future directions of AI in food safety detection are summarized.

For the transformation needs of the food industry 4.0, the breakthrough direction of AI technology will mainly focus on three dimensions. Technically, the popularity of edge computing and lightweight models will reduce the hardware cost and compress the cost of equipment to below USD 1,500, benefiting small production entities [199,200,201]. The development of multimodal large models will drive innovation in data fusion, integrating satellite remote sensing, production line sensors, and consumer data to build a global risk assessment system from the composition safety of raw materials to table nutrition. At the governance level, it is necessary to establish algorithm authentication standards and data sharing mechanisms to achieve global data compliance flow. At the industrial level, industry–university–research collaborative innovation will accelerate the adaptation of technology and build a quality control ecosystem covering the whole chain through smart devices, cloud services, and blockchain [202,203,204].

In the next decade, AI-driven food safety management will show three major development trends. First, the miniaturization of intelligence detection equipment will be achieved through a combination of nano sensors and AI to achieve the real-time monitoring of trace pollutants. Second, the decision-making model is transparent, and XAI technology will reveal the correlation of pollution events through causal reasoning. Thirdly, the ecological management system and the establishment of a transnational food safety database will enhance the coordination of global risk prediction [205]. These technological improvements will not only improve the level of food safety but also restructure the food trust system. By analyzing the full life cycle data of every food product with an AI system, personalized nutrition advice and agricultural production data can be intelligently matched. Then, food safety management will shift from risk prevention and control to value creation, finally forming a new food ecology with data-driven production, intelligent security, and healthy technology.

AI technology is becoming the core driving force of global food safety governance, but its development needs a three-in-one support system of technology, ethics, and policy [206]. By continuing to break through key technical bottlenecks such as data standardization, model interpretability, and the weight of equipment and improving cross-field collaboration mechanisms and legal and regulatory frameworks, AI will eventually achieve a paradigm shift from passive supervision to active prevention, provide intelligent solutions for global food safety, and allow the food industry to progress in an efficient, safe, and sustainable direction [207,208,209].

## 5. Conclusions

In the context of the deep integration of globalization and digitalization, AI technology is reshaping the food safety governance paradigm through full-chain penetrating supervision, and its core value is to achieve accurate control from farm to fork through data-driven, intelligent decision-making. In the process of source control, AI integrates spectral analysis, machine vision, and DL algorithms to build a dynamic monitoring system to realize detect diseases and pests, pesticides, veterinary drug residues, and heavy metal pollution. Through multi-modal data fusion technology, the supply chain can improve the sorting efficiency and cold chain monitoring accuracy and realize the quality traceability of the whole process. The processing process relies on intelligent sensors and neural network models to monitor production parameters in real time and predict the migration amount of harmful ingredients in packaging materials. On the consumer side, colorimetric sensing and edge computing technology are used to realize the second-level evaluation of food freshness and early warning of spoilage. A reliable traceability system jointly built by blockchain and AI ensures that data cannot be tampered with and enhances the transparency of the supply chain. A personalized, intelligent service system realizes accurate nutrition matching, food demand forecasting, and personalized meal matching through multi-source data fusion. AI technology has not only brought revolutionary changes to food safety governance but has also provided consumers with safer, healthier, and personalized food choices.

Although AI shows great potential in the field of food safety, its application also faces technical obstacles such as insufficient data standardization, a lack of algorithm interpretability, the heterogeneity of food samples leading to limited model generalization ability, the difference in detection tools increasing the problem of model migration, and the high cost of intelligent transformation restricting the landing of technology. In the future, with the continuous progress of technology, edge computing will promote the miniaturization of detection equipment, achieve real-time monitoring cost compression, integrate multi-modal large models and multi-source data such as spectra and images, build a global risk assessment system, and frontier technologies such as quantum sensing to break through the detection sensitivity limit. AI is expected to play a more important role in the field of food safety, promoting food safety management from risk prevention and control to value creation, ultimately forming a new food ecology, with data leading production changes and intelligent security defense lines and technology helping to upgrade health. This will provide intelligent solutions for global food safety and allow the food industry to progress in an efficient, safe, and sustainable direction.

## Figures and Tables

**Table 1:** Comparison of the application of AI technology in food safety.

AI Branch	Advantages	Disadvantages	Applicable Scenarios
Supervised learning	Easy to explain, suitable for small samples	Depends on labeled data, limited ability to extract complex features	Classification tasks, such as disease recognition, pesticide residue detection
Unsupervised learning	No need for labeled data, ability to discover hidden patterns	Poor result interpretability, relies on assumptions about data distribution	Anomaly detection, food sorting
Semi-supervised learning	Combine limited labeled data with abundant unlabeled data to cut labeling costs.	High model complexity, need to balance the impact of labeled and unlabeled data	Small sample scenarios
Deep learning	Automatic feature extraction, capable of handling high-dimensional data	High demand for computing resources, poor interpretability	Image recognition, such as meat freshness, pathogen detection
Internet of things	Real-time monitoring, fusion of multi-source data	Data heterogeneity, transmission delay	Warehouse environment monitoring
Blockchain	Data immutability, enhanced traceability transparency	High storage costs, difficulty in collaborative governance	Full-chain traceability, production–distribution–consumption

**Table 2:** Application of AI-based inspection methods in food source quality control, intelligent sorting before preprocessing, and food storage safety.

Foods	Detection Methods	ML Algorithms	Model Performance	Ref.
Nut	Machine vision	MRFCN, CNN	RS: 99.4% Acc., ID: 96.1% Acc.	[100]
Can	Machine vision	ERC, MSRD	MSRD: 99.48% Acc.	[107]
Pear	X-ray tomography	SVM	SVM: 92.2% Acc.	[108]
Fish	Colorimetry	CNN, VGG16	CNN: 96.2% Acc.	[115]
Cassava	Machine vision	CNN	CNN: 93% Acc.	[123]
Tomato	Machine vision	YOLOv8	Test confidence: 87%	[124]
Spinach	SERS	Transformer	Transformer: 98.4% Acc., MAE = 0.966	[130]
Fruits	SERS	CNN, SVM, RF	CNN: 99.62% Acc.	[131]
Water	Fluorescence sensor	Aug-MLP, KNN, SVM, GNB, RF	Aug-MLP: 83.1% Acc.	[139]
Corn	Genomics technology	Bayes, rrBLUP, RF	rrBLUP: ACC = 0.89, MAE = 0.0037	[140]
Mutton	Hyperspectral imaging	CNN-SSAE	CNN-SSAE: 93.65% Acc.	[143]
Crop	Data-driven	GA, BP	GA: 98.18% R^2^	[144]
Lettuce	Sensor data	SAC	32.34% energy saved	[145]
Nut	Spectrum	ELM, SVM, LDA, QDA, PLS-DA	SVM: 5.54% LR, 98% CR	[146]
Meat	Fluorescence spectrum	LDA, QDA	Linear, R^2^ = 0.99	[147]
Chicken	SERS, gas array sensor	PCA, LDA	LDA: 96.9% Acc.	[148]
Chicken	IR	RSDE	RSDE: 95% Acc.	[149]
Crop	Data-driven	BP, SVM	Error: 15%~20%	[150]
Crop	Data-driven	XGBoost	SFMA = 0.61, TFMA = 0.51	[151]
Crop	Machine vision	CNN	CNN: 83.8% Acc.	[152]
Fish	E-nose	BP, GA-BP, RBFNN, ELM	RBF: MAE = 0.118, R^2^ = 0.9994	[153]
Fish	Gas sensor	RF, SVM, DNN	RF, SVM: 95.83% Acc.	[154]

The abbreviations in Table 2 are explained as follows. **MRFCN**: multi-scale residuals full convolutional network; **CNN**: convolutional neural network; **RS**: region segmentation; **Acc.**: accuracy; **ID**: impurity detection; **ERC**: entropy rate clustering; **MSRD**: multi-scale ridge detection; **SVM**: support vector machine; **VGG16**: visual geometry group 16-layer network; **YOLOv8**: You Only Look Once version 8; **SERS**: surface-enhanced Raman spectroscopy; **MAE**: mean absolute error; **MSE**: mean square error; **RF**: random forest; **Aug-MLP**: enhanced multilayer perceptron; **KNN**: K-nearest neighbors; **GNB:** gaussian naive Bayes; **rrBLUP**: ridge regression best linear unbiased prediction; **ACC**: average correlation coefficient; **CNN-SSAE**: convolutional neural network-stacked sparse auto-encoder; **GA**: genetic algorithm; **BP**: backpropagation neural network; **R^2^**: determination coefficient; **SAC**: soft actor–critic; **ELM**: extreme learning machine; **LDA**: latent Dirichlet allocation; **QDA**: quadratic discriminant analysis; **PLS-DA**: partial least squares discriminant analysis; **LR**: loss rate; **CR**: classification rate; **PCA**: principal component analysis; **IR**: infrared radiation; **RSDE**: random subspaces discriminative ensemble; **XGBoost**: eXtreme gradient boosting; **SFMA**: seven F1 macro average; **TFMA**: three F1 macro average; **E-nose**: electronic nose; **GA-BP**: genetic algorithm–backpropagation; **RBFNN**: radial basis function neural network; **DNN**: deep neural network.

**Table 3:** AI-based intelligent control in food processing, rapid detection, trusted traceability of blockchain +AI, and personalized intelligent services.

Foods	Detection Methods	ML Algorithms	Model Performance	Ref.
Condiment	Machine vision	CNN	CNN: 95.71% Acc.	[156]
Functional food	Ion trap analysis	SVM	SVM: 99.78% sensitivity	[155]
Cereal	Data-driven	RF	RF: AUC = 0.96	[158]
Fatty food	GC-MS	RBF ANN	R^2^ 0.95, MSE = 0.046	[159]
Milk	Line image sensor	XGBoost	XGBoost 96% Acc.	[165]
Pork	Fluorescence detection	RBFNN	RBFNN: 100% Acc.	[166]
Milk	Fluorescence sensor	ANN	ANN: 93.8% Acc.	[167]
Fish	Colorimetry	RF	RF: 98.8% Acc.	[169]
Chicken	Colorimetry	ANN	ANN: R^2^ = 0.9946	[170]
Asparagus	FT-NIS	SVM	SVM: 89% Acc.	[178]
Liquor	GC × GC/TOF-MS	PCA, SVM, RF	SVM: 97.67% Acc., RF: 95.36% Acc.	[179]
Feed	Data-driven	NN, Non-NN	NN: 86.02% Acc.	[181]
Noodles	SEM, TD-NMR	KPCA	/	[183]
Meat	Data-driven	RF, LSTM, Transformer	RFW 4% to 52%, RUN 3% to 16%	[184]
Food composition	Data-driven	L1-RLR	L1-RLR: 84% Acc., AUC = 0.92	[185]
Micronutrient	Data-driven	RF, GBM, SVM, KNN	Accuracy >80%	[186]

The abbreviations in Table 3 are explained as follows. **AUC**: area under curve; **GC-MS**: gas chromatography–mass spectrometry; **RBF ANN**: radial basis function artificial neural network; **ANN**: artificial neural network; **FT-NIS**: Fourier transform near-infrared spectroscopy; **GC × GC/TOF-MS**: full two-dimensional gas chromatography–time-of-flight mass spectrometry; **DT**: decision tree; **NonNN**: non-neural network; **SEM**: scanning electron microscope; **TD-NMR**: time domain NMR; **RFW**: reduced food waste; **RUN**: reduced unmet needs; **KPCA**: kernel principal component analysis; **LSTM**: long short-term memory; **L1-RLR**: L1-regularized logistic regression; **GBM**: gradient boosting machine.

**Table 4:** Challenges and future directions of AI in food safety detection.

Dimensionality	Challenges	Future Direction
Technical level	Cross-modal fusion is difficult. The model is not robust and interpretable enough. Real-time, small sample, multitasking bottleneck.	Cross-modal framework. Robust algorithm, explainable AI technology. Lightweight model, multi-learning, multitask optimization.
Data level	■ Uneven quality, privacy barriers, difficult to adapt dynamic data. ■ Multi-source integration, labeling cost, spatio-temporal deviation problems.	■ Data enhancement, privacy computing, dynamic graph. ■ Cross-modal pre-training, active learning, domain adaptation, time series modeling.
Regulation and ethics	◆ Lack of standards, fuzzy responsibility. ◆ Hidden fairness, human–machine decision conflict. ◆ Judicial effectiveness dispute, technology dependence risk.	◆ Standardization construction, responsibility sharing. ◆ Fairness algorithm, man–machine coordination mechanism. ◆ Judicial certification, skills maintenance system.
Application and industry	🞇 High deployment cost and talent shortage. 🞇 Equipment compatibility, whole chain coordination, and compliance transformation are difficult.	🞇 Lightweight solutions, interdisciplinary talents. 🞇 Standardized interface, whole chain modeling. 🞇 Compliance tool development.
Environmental sustainability	★ High computing power consumption, electronic waste pollution. ★ Excessive consumption of resources.	★ Green AI technology, circular economy model. ★ Sustainability optimization model.
Global perspective	❖ Regional differences, barriers to mutual recognition of standards. ❖ Language, culture, geopolitical influence.	❖ Regional adaptation model, international coordination mechanism. ❖ Multi-language processing, localization technology ecology.

## Data Availability

No new data were created or analyzed in this study. Data sharing is not applicable to this article.

## Abbreviations

The following abbreviations are used in this manuscript:

AD	anomaly detection
Acc.	accuracy
AE	automatic encoder
ACC	average correlation coefficient
AI	artificial intelligence
ANN	artificial neural network
AUC	area under curve
Aug-MLP	enhanced multilayer perceptron
BP	backpropagation
CA	cluster analysis
CNN	convolutional neural network
CNN-SSAE	convolutional neural network-stacked sparse auto-encoder
CTM	co-training model
DL	deep learning
DNN	deep neural network
DR	dimension reduction
DT	decision tree
ELM	extreme learning machine
E-nose	electronic nose
ERC	entropy rate clustering
FT-NIS	Fourier transform near-infrared spectroscopy
GA	genetic algorithm
GA-BP	genetic algorithm–backpropagation
CR	classification rate
GBM	gradient boosting machine
GC × GC/TOF-MS	full two-dimensional gas chromatography–time-of-flight mass spectrometry
GC-MS	gas chromatography–mass spectrometry
GelMA	gelatin–methylacrylyl
GM	generative model
GNB	gaussian naive Bayes
ID	impurity detection
IR	infrared radiation
K-means	K-means clustering algorithm
KNN	K-nearest neighbor
KPCA	kernel principal component analysis
L1-RLR	L1-regularized logistic regression
LR	loss rate
LDA	linear discriminant analysis
LR	linear regression
LSTM	long short-term memory
MAE	mean absolute error
ML	machine learning
MRFCN	multi-scale residuals full convolutional networks
MSE	mean square error
MSRD	multi-scale ridge detection
NonNN	non-neural network
OFX	ofloxacin
PCA	principal component analysis
PFALs	plant factories with artificial lighting
PHI	post-harvest interval
PLS-DA	partial least squares discriminant analysis
QDA	quadratic discriminant analysis
R^2^	determination coefficient
RBF	radial basis function
RBF ANN	radial basis function artificial neural network
RBFNN	radial basis function neural network
ResNet18	residual network 18
RFW	reduced food waste
RUN	reduced unmet needs
RF	random forest
RNN	recurrent neural network
rrBLUP	ridge regression best linear unbiased prediction
RS	region segmentation
RSDE	random subspaces discriminative ensemble
SAC	soft actor–critic
SEM	scanning electron microscope
SERS	surface-enhanced raman spectroscopy
SFMA	seven F1 macro average
STM	self-training model
SVM	support vector machine
TD-NMR	time domain NMR
TSVM	transduction SVM
TFMA	three F1 macro average
VGG16	visual geometry group 16-layer network
VOCs	volatile organic compounds
XAI	explainable AI
YOLOv8	You Only Look Once Version 8
